# Impact of molar teeth distalization with clear aligners on occlusal vertical dimension: a retrospective study

**DOI:** 10.1186/s12903-019-0880-8

**Published:** 2019-08-13

**Authors:** Silvia Caruso, Alessandro Nota, Shideh Ehsani, Elena Maddalone, Kenji Ojima, Simona Tecco

**Affiliations:** 1 Department of Life, Health and Environmental Sciences, University of L’Aquila, Piazzale Salvatore Tommasi 1, 67100 L’Aquila, Coppito Italy; 2 Dental School, Vita-Salute University and IRCCS San Raffaele Hospital, Via Olgettina, 58, 20132 Milan, Italy; 3 Private Practice of Orthodontics, Tokyo, Japan

**Keywords:** Malocclusion, angle class II, Vertical dimension, Tooth movement techniques, Orthodontic appliances, removable

## Abstract

**Background:**

A common strategy in the non-extraction treatment of Class II molar relationship is maxillary molar distalization, which could increase lower face height and cause clockwise mandibular rotation. The aim of this retrospective study was to analyse the effects on vertical dentoskeletal dimension of young adults treated with sequential distalization with orthodontic aligners.

**Methods:**

Lateral cephalometric radiographs of 10 subjects (8 females 2 males; mean age 22.7 ± 5.3 years) treated with upper molars sequential distalization with orthodontic aligners (Invisalign, Align Technology, San Josè, California, USA) were analyzed.

**Results:**

No statistically significant difference was observed for the primary outcome SN-GoGn between T0 and T1 and it was recorded a mean variation of 0.1 ± 2.0 degrees. Statistically significant differences were found in the linear position of the upper molars (6-PP, 7-PP) the molar class relationship parameter (MR) and the upper incisive inclination (1^PP) with at least *p* < 0.01.

**Conclusions:**

Upper molar distalization with orthodontic aligners guarantee an excellent control of the vertical dimension representing an ideal solution for the treatment of hyperdivergent or openbite subjects. It also allows an excellent control of the incisal torque without loss of anchorage during the orthodontic procedure.

## Background

One of the most common strategies applied in the non-extraction treatment of Class II molar relationship is maxillary molar distalization. The major indication are patients with maxillary dentoalveolar protrusion or minor skeletal discrepancies [1, 2].

Since 1950’s headgear has been the most frequently used appliance for maxillary molar distalization. Unfortunately this appliance requires considerable patient compliance [3, 4] so several alternative intraoral methods had been proposed to reduce or cut out patient’s cooperation [5, 6]. Despite the effectiveness of many of these appliances clinicians must consider many side-effects: increase in lower face height, clockwise mandibular rotation, extrusion of first premolars, undesirable tipping of the maxillary molars and loss of anterior anchorage during distalization [1, 7–10]. Most of these side effects involve an increase of the vertical dimension of the treated subjects, keeping this treatment procedure generally contraindicated in hyperdivergents [2, 11].

In the last decades, the orthodontic treatment with removable clear aligners has become an increasingly common choice because of the growing number of adult patients that ask for aesthetic and comfortable alternatives to conventional fixed appliances [12, 13]. Clear aligners are based on computer aided design procedures. The orthodontic treatment with the Invisalign (Align Technology, San Josè, California, USA) system is a digitized process that starts from the acquisition of a 3D model of the dental arches allowing the planning of teeth movements with a proper software. The aligner allows the control of 3D movements by holding teeth on all the surfaces (vestibular, palatal-lingual and occlusal) and applying proper forces thanks to attachments of different size and shape and other specific features.

Aligners can also provide a class II correction by a sequential maxillary molar distalization [14, 15] with a high predictability (88%) of the distalization movement of upper molars if supported by the presence of attachments on the tooth surface assessed by Simon et al. [16, 17]. Ravera et al. [15] showed that clear aligners are suitable for distalizing maxillary up to 2-3 mm without significant mesiodistal tipping movement, and it seems that this result could be improved if combined with photobiomodulation or other acceleration tooth movement systems [18–20].

The aim of this retrospective study was to analyze the effects of class II treatment by sequential distalization with orthodontic aligners on vertical dentoskeletal dimension.

## Methods

### Subjects and procedure

**Fig. 1:**
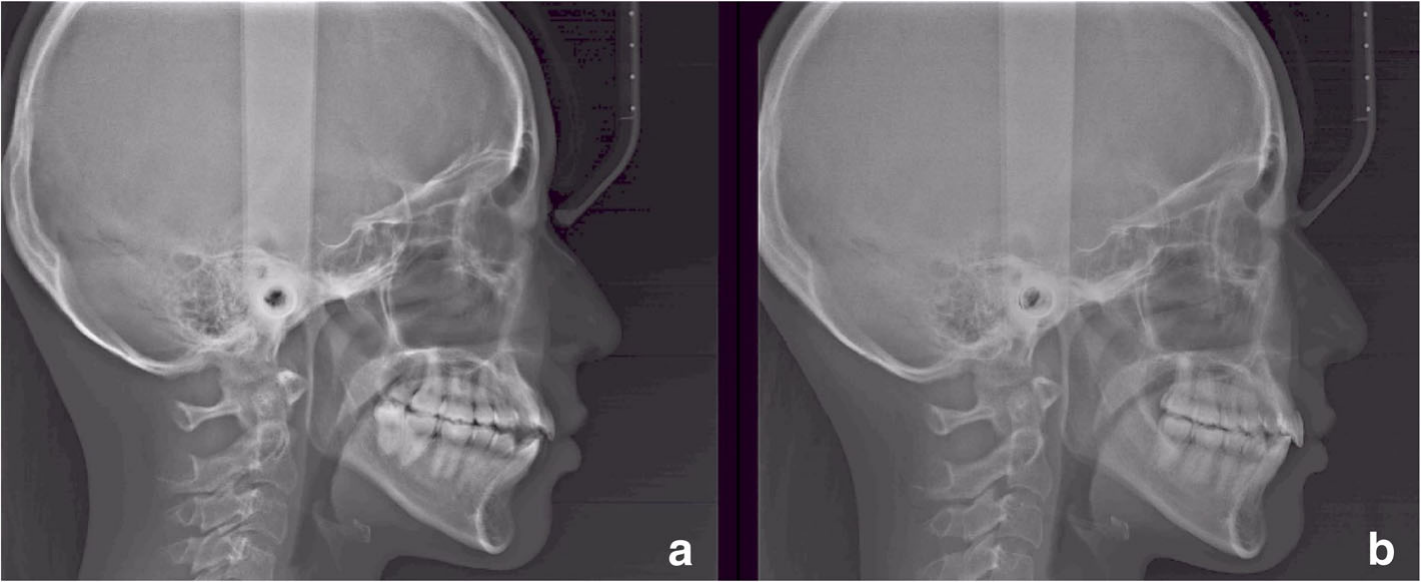
**a-b**: Lateral cephalometric radiographs of a patient, before the orthodontic treatment with sequential distalization (**a**) and after treatment (**b**)

This retrospective study analysed lateral cephalometric radiographs of a sample of 10 subjects (8 females 2 males; mean age 22.7 ± 5.3 years) treated with sequential distalization with orthodontic aligners (Invisalign, Align Technology, San Josè, California, USA). Figure 1 shows the lateral cephalometric radiographs of a patient included in this study. The retrospective study was ethically approved by the Institution, the procedures were in accordance with the declaration of Helsinki and all the subjects signed a consent form. Bilateral molar class II or end-to-end molar relationship, absence of mesial rotation of the upper first molars, mild or light crowding in the upper arch, absence of periodontal disease, absence of previous prosthodontic treatments of the upper molars, good compliance during the treatment, good quality and definition of the radiographs were the study inclusion criteria. All the subjects that satisfied the inclusion criteria were included in the study and were successfully treated even if the treatment success wasn’t an inclusion criterion. The mean treatment time was of 1.9 ± 0.5 years. Four subjects were excluded from the initial study sample of 14 subjects because they didn’t match with the inclusion criteria.

Lateral cephalograms in habitual occlusion were considered for the study. Cephalometric head films were collected at the beginning (T0) at the end of treatment (T1) with orthodontic aligners.

**Fig. 2:**
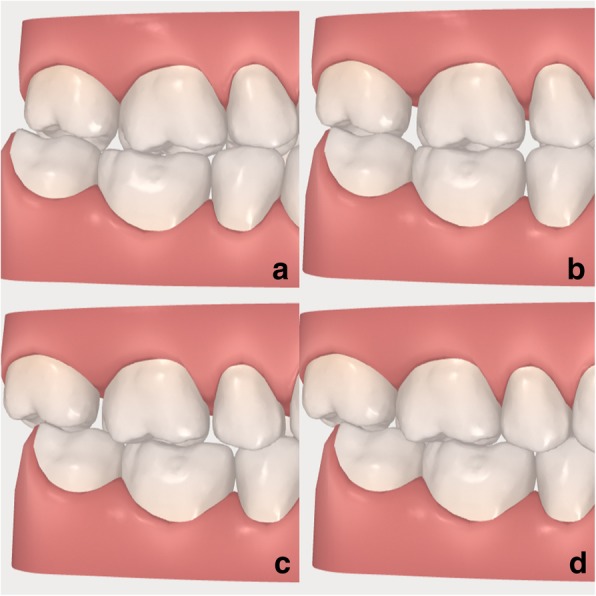
**a-d**: Sequence of tooth movement with distalization of the upper teeth, from **a** to **d**. Frames extracted by a ClinCheck® (Align Technology, San Josè, California, USA)

The treatment of sequential upper arch distalization (Fig. 2) was performed by the same expert operator (K.O.) as proposed by Align Technology and described by Ravera et al. [15] using II class elastics and rectangular vertical attachments on the upper molars and premolars.

Radiographs were manually traced by the same expert operator (S.E.) blinded about the study. A total of fourteen cephalometric parameters (5 linear, 9 angular) were measured and recorded for each cephalogram, afterwards the relationship between the posterior facial height and the anterior facial height were calculated.

SN-GoGn (°) was considered as primary outcome. [21] It shows the impact of the orthodontic procedure on the sagittal vertical dimension of the samples.

### Intra-observer method error

In order to verify the method error, ten lateral cephalometric radiographs underwent to the same cephalometric analysis two times by the same operator, at a distance of about 2 weeks. Applying the Dahlberg’s formula, the method error resulted lower than the standard deviation observed in the whole sample for the variable. For the primary outcome the measured method error was 0.98°. The intraclass correlation coefficient (ICC) was also calculated, for the primary outcome, obtaining a value of 0.99.

### Statistical analysis

Descriptive statistics were calculated for each variable of recorded data.

The normality assumption of the data was confirmed by the Shapiro-Wilk test. Thus, the differences between before (T0) and after treatment (T1) were compared with the paired-t test. The level of significance was set at *P* < 0.05.

## Results

**Fig. 3:**
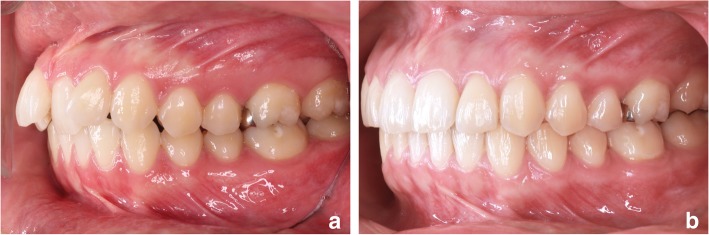
**a-b**: Lateral intra-oral view of a patient, before the orthodontic treatment (**a**) and after treatment (**b**)

Descriptive data, means and standard deviation (SD), of the recorded parameters are reported in Table 1. Figure 3 shows a lateral intra-oral view of one of the treated patients, before and after treatment.

**Table 1:** Descriptive data and statistical analysis of the differences between T0 and T1

	T0		T1		Student T Test
	MEAN	SD	MEAN	SD	Sig.
SNA (°)	82.4	4.7	83.0	4.9	0.559
SNB (°)	79.0	4.9	78.7	4.9	0.403
ANB (°)	3.4	3.3	4.3	3.2	0.195
SN^GoGn (°)	35.6	6.9	35.4	8.4	0.445
SN^fOP (°)	18.9	4.1	20.6	6.3	0.122
SN^PP (°)	7.3	6.1	6.3	5.7	0.309
6-PP (mm)	25.0	3.0	23.0	3.0	0.000****
6^PP (°)	81.2	3.5	79.9	4.4	0.220
7-PP (mm)	16.0	3.0	13.0	3.0	0.000****
7^PP (°)	81.7	5.6	82.3	4.3	0.352
1^PP (°)	118.3	6.6	104.8	10.9	0.006***
MR (mm)	3.1	1.4	1.2	0.6	0.000****
S-Go (mm)	68.0	6.1	68.0	6.8	0.476
N-Me (mm)	109.0	6.0	108.9	6.2	0.438
S-Go/N-Me	0.62	0.05	0.63	0.06	0.421

* = *P* < 0.05; ** = *P* < 0.01; *** = *P* < 0.001; **** = *P* < 0.0001

No statistically significant difference was observed for the primary outcome SN-GoGn between T0 and T1 and it was recorded a mean variation of 0.1 ± 2.0 degrees. Similarly no statistically significant difference was observed for the linear measurements of vertical dimension (S-Go;N-Me).

Statistically significant differences were found for the linear position of the upper molars (6-PP, 7-PP) the MR parameter and the upper incisor inclination (1^PP) with at least *p* < 0.01.

No significant variations were observed for the other cephalometric parameters analysed.

## Discussion

In literature it was observed that different orthodontic appliances caused undesired effects on the upper molars distalization procedure and on the sagittal vertical pattern as clockwise rotation of the mandibular plane and increase in the anterior facial height [22–26]. This finding implied a contraindication of the upper molar distalization in hyperdivergent subjects.

The present retrospective study analysed the sagittal vertical dimension changes associated with successful orthodontic treatment of subjects with second molar class by sequential upper molar distalization performed with clear aligners. A previous study showed a high predictability of clear aligners in performing the upper molars distalization movement with absence of distal tipping [15].

Results indicated that there were no changes in the subject divergence by observing variations of the SN-GoGn angle lower than 1°. The present findings suggest that clear aligners allow a good control of mandibular divergence during molar distalization. These results are in accordance with what reported by Ravera et al. [15] as a secondary outcome of their study.

Similarly, it was observed a significant distal movement of the upper molars (and the related correction in molar relationship) with absence of distal tipping, confirming the capability of performing a distal body movement of the upper molars by clear aligners with a control of the vertical dimensions the opposite of what reported by previous authors [22–26] with other orthodontic appliances.

No significant rotations of the maxillary and functional occlusal plane were observed. No significant changes were observed in the sagittal position of mandible and maxilla, in contrast to what reported by Ravera et al. [15] that showed a significant reduction of the ANB angle.

Previous studies showed a control of the vertical dimension during distalization with pendulum appliance properly activated by expert operators [2, 22, 24]. Recent review indicates a molar distal tipping that range between 8.4° and 14.5°, much higher than what reported by the present study (mean tipping of 1.3°), furthermore a trend to an anterior anchorage loss was observed with pendulum appliance if bone anchorage was not applied [25, 26].

No anchorage loss was observed on upper incisors that had a significant mean reduction of their inclination of 13.2° showing a torque control much higher than what reported by Ravera et al. [15].

Looking at the results of this study, the upper molar distalization performed with clear aligners seems to overcome various side effects related with this orthodontic procedure typically observed with other appliances in previous studies [1, 7–10] and seems to allow a predictable distal body movement of upper molars [15–17] with a control of the vertical dimension and of the incisal torque. This could be related with the aligner design, that allows the control of 3D movements by holding teeth on all the surfaces (vestibular, palatal-lingual and occlusal) and applying proper forces thanks to properly digitally planned attachments.

Consequently, orthodontic aligners could represent an effective alternative for upper molar distalization especially in hyperdivergent or openbite subjects at least for distal molar movements up to 2–3 mm. Further studies should be conducted on distal molar movements higher than 2–3 mm and on hyperdivergent subjects.

At the authors’ knowledge this is the first study that analysed as a primary outcome the effects of the upper molar distalization orthodontic technique with clear aligners on the vertical dimension of subjects with molar class II malocclusion.

### Limitations of the study

Limitations of this study are the low sample size and the limited mean amount of distal movement that should be increased in future studies to confirm the control of the vertical dimension. Furthermore, the retrospective design should be replaced by a longitudinal design in order to reduce the risk of bias.

## Conclusions

Upper molar distalization with orthodontic aligners properly digitally planned by the orthodontist seems to allow a good control of the vertical dimension. A satisfactory control of the incisal torque without loss of anchorage during the orthodontic procedure was also observed.

Further studies should be performed to confirm the results of the present study and analyse if the upper distalization with orthodontic aligners could represent an effective alternative for the treatment of class II subjects even with hyperdivergent or openbite skeletal patterns.

## Data Availability

The data that support the findings of this study are available from the archive of the University of L’Aquila, but restrictions apply to the availability of these data, which were used under permission and consent for the current study, and so are not publicly available. Data are however available from the authors upon reasonable request and with permission of the patients and the Ethic Committee of the University of L’Aquila.

## Abbreviation

MR: Molar Relationship
